# Efficacy and Safety of Deferred Stenting in Geriatric Patients with STEMI and High Thrombus Burden

**DOI:** 10.31083/j.rcm2503088

**Published:** 2024-03-05

**Authors:** Ruifang Liu, Fangxing Xu, Tongku Liu, Yujie Zhou, Xiaofan Wu

**Affiliations:** ^1^Department of Cardiology, Beijing Anzhen Hospital, Capital Medical University, Beijing Institute of Heart Lung and Blood Vessel Disease, 100029 Beijing, China; ^2^The Center of Cardiology, Affiliated Hospital of Beihua University, 132011 Jilin, Jilin, China

**Keywords:** geriatric patients, ST-segment elevation myocardial infarction, thrombus burden, deferred stenting, efficacy, safety

## Abstract

**Background:**

Deferred stenting has been recognized as beneficial for patients with acute ST-segment elevation myocardial infarction (STEMI) accompanied by a high thrombus burden. Nevertheless, its efficacy and safety specifically in geriatric STEMI patients remain to be elucidated. This study aims to bridge this knowledge gap and assess the potential advantages of deferred stenting in an older patient cohort.

**Methods:**

In this study, 208 geriatric patients (aged ≥80 years) with STEMI and a high thrombus burden in the infarct-related artery (IRA) were enrolled. They were categorized into two groups: the deferred stenting group, where stent implantation was conducted after 7–8 days of continuous antithrombotic therapy, and the immediate stenting group, where stent implantation was performed immediately.

**Results:**

In the deferred stenting group, the stents used were significantly larger in diameter and shorter in length compared to those in the immediate stenting group (*p* < 0.05). This group also exhibited a lower incidence of distal embolism in the IRA, and higher rates of the thrombolysis in myocardial infarction (TIMI) blood flow grade 3 and myocardial blush grade 3 (*p* < 0.05). Additionally, the left ventricular ejection fractions at the 1-year follow-up were significantly higher in the deferred stenting group than in the immediate stenting group (*p* < 0.05). The rate of the major adverse cardiac events in the deferred stenting group was significantly lower than in the immediate stenting groups (*p* < 0.05).

**Conclusions:**

Deferred stenting for geriatric patients with STEMI and high thrombus burden demonstrates significant clinical benefits. This approach not only reduces the incidence of distal embolism in the IRA, but also enhances myocardial tissue perfusion and preserves cardiac ejection function. Moreover, deferred stenting has proven to be safe in this patient population, indicating its potential as a preferred treatment strategy in such cases.

## 1. Introduction

The primary goal in acute ST-segment elevation myocardial infarction (STEMI) management is the immediate opening of the infarct-related artery (IRA) [1]. This critical action aims to reestablish forward blood flow, salvage the jeopardized myocardium, and preserve cardiac heart function [1]. Primary percutaneous coronary intervention (PCI) with stent implantation is currently the standard of care for STEMI patients [2, 3, 4]. In geriatric patients with STEMI and high thrombus burdens (thrombus score ≥4), deferred stenting has shown favorable outcomes [5]. This is especially the case after restoring blood flow to the IRA following the thrombolysis in myocardial infarction (TIMI) blood flow grade 2 or 3 through emergency percutaneous transluminal coronary angioplasty (PTCA) and/or thrombus aspiration [5].

Current guidelines do not recommend the routine use of delayed stenting for all STEMI patients, necessitating alternative treatment strategies [2, 3]. Large-sample randomized trials have demonstrated that routine use of delayed stenting does not benefit this subset of the STEMI population [6]. Conversely, the advantage of delayed stenting in selected STEMI patients with high thrombotic burden is supported by most observational studies [7, 8, 9]. However, there is a notable scarcity of research on the efficacy and safety of deferred stenting in geriatric patients (aged ≥80 years) with STEMI and a high thrombus burden. This study seeks to address this gap by comparing the outcomes of deferred versus immediate stenting in this specific patient group.

## 2. Methods

### 2.1 Cases and Grouping

A total of 208 geriatric patients (age ≥80 years old) with STEMI and a high thrombus burden (thrombus score ≥4) in the IRA were retrospectively analyzed. These patients who underwent PCI (within 12 hours from onset of symptoms to balloon dilatation) were treated at Beijing Anzhen Hospital and Affiliated Hospital of Beihua University, China from January 2015 to January 2021. The research subjects were registered in the two catheterization laboratories. The recalculated score (rescore) of residual thrombus burden in the IRA of those patients was performed. Even after achieving stable TIMI grade 2–3 blood flow through emergency PTCA (using 1.5 × 15 mm or 2.0 × 20 mm balloon) and/or thrombus aspiration, their recalculated thrombus scores remained ≥4.

**Fig. 1.**
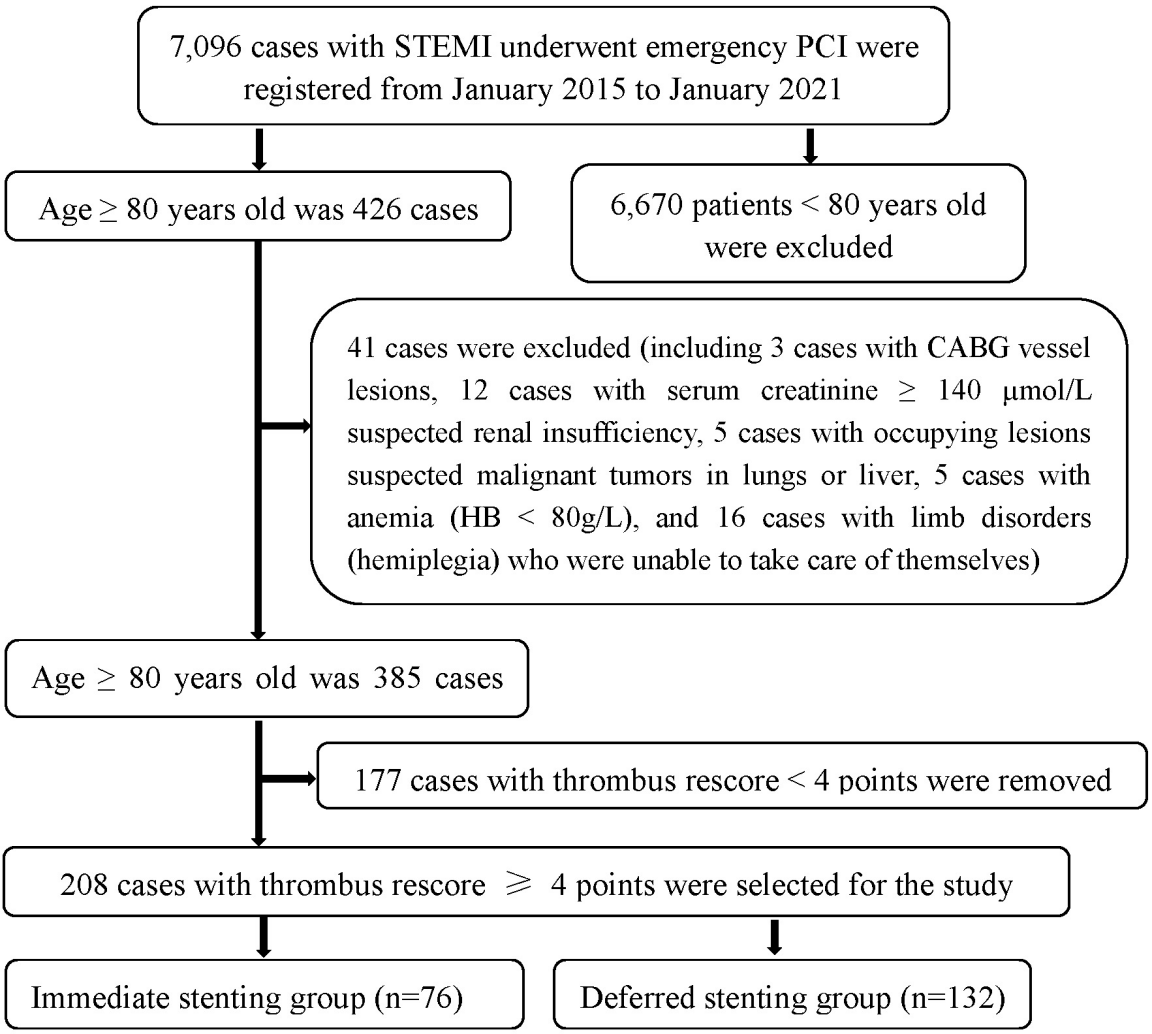
**Inclusion flow chart of patients**. CABG, coronary artery bypass grafting; HB, hemoglobin; STEMI, ST-segment elevation myocardial infarction; PCI, percutaneous coronary intervention.

The patients were categorized into two groups: a deferred stenting group (132 cases) and an immediate stenting group (76 cases). This categorization was based on the timing of stent implantation relative to the achievement of stable blood flow (Refer to Fig. 1). In the immediate stenting group, drug-eluting stents were implanted right after restoring stable flow. In contrast, the deferred stenting group received drug-eluting stents only after 7–8 days (average 7.11 ± 0.32 days) of ongoing antithrombotic treatment (including dual antiplatelet and anticoagulant therapy). STEMI diagnosis met the diagnostic criteria described in ACC/AHA/SCAI guidelines [10].

### 2.2 Inclusion Criteria and Exclusion Criteria

The inclusion criteria were: (1) Age ≥80 years; (2) Diagnosis of STEMI confirmed by chest pain symptoms, specific characteristics of electrocardiogram, elevated troponin I, findings of coronary angiography (CAG), and meeting the diagnostic criteria described in ACC/AHA/SCAI guidelines [10]; (3) A time from the onset of symptoms to balloon dilatation of within 12 hours; (4) IRA thrombus reclassification (rescore) of 4 or higher after thrombus aspiration and PTCA; (5) The patients or their legal representative agreed to PCI and signed the informed consent form.

The exclusion criteria were: (1) STEMI caused by coronary artery bypass grafting vessel lesions; (2) Non-ST segment elevation acute myocardial infarction; (3) IRA thrombus rescore of less than 4 after thrombus aspiration and/or PTCA; (4) Major surgery with significant trauma in the past week; (5) Renal failure; (6) Platelet counts <100 × ${\mathrm{10}}^{9}$/L; (7) Diagnosis of an uncontrolled infectious diseases (local or systemic infection); (8) Advanced stage of malignant tumor; (9) Diagnosis of a blood system diseases, or moderate to severe anemia (hemoglobin ≤80g/L); (10) Severe contrast agent allergy; (11) Refusal of PCI treatment.

### 2.3 PCI Methods

Patients were treated with 300 mg aspirins and 600 mg clopidogrel orally administered within either 6 hours of PCI or immediately after PCI. Heparin sodium 100 U/kg was injected intravenously during PCI to maintain the activated clotting time of whole blood (ACT) from 350 s to 500 s. CAG and PCI was performed according to the conventional method. When the IRA was opened by PTCA (with 1.5 × 15 mm or 2.0 × 20 mm balloon) and/or thrombus aspiration (Manual thrombus aspiration with Export AP Catheter, Medtronic Inc. 710 Medtronic PKWY., N.E, Minneapolis, MN 55432- 5604, USA), and the blood flow restored to TIMI grade 2–3, tirofiban (platelet glycoprotein Ⅱb/Ⅲa receptor antagonist) 10 µg/kg was routinely injected into the coronary artery by a guiding catheter. After the stable blood flow was observed for 10 min, the strategy of immediate stenting or deferred stenting was selected by the interventional cardiologist. The stent was immediately implanted in the immediate stenting group. In the delayed stenting group, stenting was performed after 7–8 days (mean 7.11 ± 0.32 days) of continuous antithrombotic (standard dual antiplatelet and anticoagulation) therapy. Heparin sodium in the deferred stenting group was injected subcutaneously at 100 U/kg every 8 hours (for 7–8 days) until the next PCI. After the stenting procedure, all patients received low molecular weight heparin (Enoxaparin sodium 1.0 mg/kg was injected subcutaneously, every 12 hours, for three days) and maintenance doses of aspirin 100 mg/d and clopidogrel 75 mg/d or ticagrelor 90 mg/twice a day (oral administration for at least one year). Angiotensin-converting enzyme inhibitors, β receptor blockers, nitrates, and statins were administrated according to the patient’s condition.

### 2.4 Data Collection and Observation Indicators

The patient data collected included age, sex, smoking history, medical history (hypertension, diabetes, hyperlipidemia, stroke, and so on), data from laboratory examination, and PCI data (including IRA distribution, thrombus burden score, time from onset of symptoms to balloon dilatation, and number, diameter, and length of stent implantation).

The observation indicators included the TIMI flow grade of IRA before and after stenting, distal embolism rate, myocardial blush grade, left ventricular ejection fractions (LVEF), and major adverse cardiac events (MACE) one year after stenting. MACE included all-cause mortality, recurrence of nonfatal myocardial infarction, target lesion revascularization (TLR), stroke, readmission for heart failure, and repeat PCI (including any unplanned revascularization of the target vessel or anyone of the right coronary artery, left anterior descending branch, and left circumflex branch) during follow-up.

### 2.5 Evaluation Criteria of the Thrombus Burden

Evaluation of the thrombus burden in IRA was performed before and after PTCA and/or thrombus aspiration. Evaluation criteria of the thrombus burden are as follows [11]: 0 point is no thrombus; 1 point defined as a fuzzy shadow; 2 points is defined as thrombus imaging in which the length is less than half of the blood vessel diameter; 3 points is defined by the presence of blood clots where the length is 1/2-2 times of the vascular diameter; 4 points is given when the diameter and the length of certain blood clots are greater than 2 times of the vascular diameter; and 5 points are given when there is a complete occlusion of the blood vessel. A patient is usually considered to have a high thrombus burden in the IRA when a thrombus score is equal to or greater than 4 points [12, 13].

### 2.6 Classification Standard of TIMI Flow Grade

TIMI flow grade in IRA was evaluated before and after PTCA and/or thrombus aspiration, and stenting. The classification standard of TIMI blood flow grade [14] is as follows: Grade 0: No blood flow perfusion; Grade 1: Micro blood flow perfusion, but the contrast agent cannot reach the distal vessels; Grade 2: Partial blood flow perfusion, but the contrast agent cannot reach the distal vessels within 3 cardiac cycles; Grade 3: Complete blood flow perfusion, and the contrast agent can reach the distal vessels within 3 cardiac cycles.

### 2.7 Evaluation of Distal Vascular Embolization

Evaluation of distal vascular embolization was performed immediately after stenting. Vascular embolism is defined as any blockage of the peripheral vascular branch of a diameter ≥1.0 mm or contrast agent retention in the distal target vessel [15].

### 2.8 Evaluation of the Myocardial Blood Perfusion

Determination of myocardial reperfusion dyeing is based on the methods of Van Hof’s myocardial blush grade (MBG) classification [16]. Grade 0 is no myocardial blush. Grade 1 is minimal myocardial blush. Grade 2 is moderate myocardial blush. Grade 3 is a normal myocardial blush. MBG was performed immediately after stenting.

### 2.9 Method and End Point of Follow-up

The follow-up was performed by outpatient, inpatient, or telephone. Follow-up measures included medical history, symptoms, physical examination, electrocardiographic examination, and echocardiographic examination. The endpoint of follow-up was the hard clinical endpoint of MACE. The follow-up ended on December 31, 2022.

### 2.10 Statistical Analysis

All data were analyzed by using the Statistical Package for Social Sciences software (SPSS) 20.0 (SPSS, version 20.0, SPSS Inc., Chicago, IL, USA). The continuous variables with normal distributions were expressed as mean ± standard deviation ($\bar{x}$ ± S). Comparisons between groups were performed using the independent Student’s *t*-test. The counting data were expressed as a percentage (%), and the chi-square (χ^2^) test was used for comparison between groups. The test level was set as a double-tail test a = 0.05, and *p* < 0.05 was statistically significant, and *p* < 0.01 was very statistically significant.

## 3. Results

### 3.1 Comparison of Baseline Data in Two Groups

A total of 208 patients recruited for this study, divided between deferred (n = 132) and immediate (76) stenting. The focus of the study was on geriatric patients, and age range varied from 80–87 years, with an average age of 83.00 ± 2.39. There were no significant differences (*p* > 0.05) in the prevalence of hypertension, hyperlipidemia, diabetes, smoking, angina pectoris, stroke, and other variables listed below between the two groups (See Table 1 for details).

**Table 1.** **Baseline characteristics of the deferred and immediate stenting groups**.

	Deferred stenting (n = 132)	Immediate stenting (n = 76)	χ^2^/*t* value	*p* value
Age (years old)	82.79 ± 2.36	83.37 ± 2.43	1.692*	0.092
Female, n (%)	54 (63.64)	39 (51.32)	3.03	0.107
Hypertension, n (%)	64 (48.48)	26 (34.21)	4.003	0.059
Hyperlipidemia, n (%)	23 (17.42)	14 (18.42)	0.033	0.856
Diabetes, n (%)	26 (19.70)	18 (23.68)	0.460	0.498
Months of diabetes	126.35 ± 28.35	130.94 ± 41.40	–0.438*	0.664
Smoking, n (%)	29 (21.97)	14 (18.42)	0.370	0.543
Stroke, n (%)	8 (6.06)	4 (5.26)	0.056	1.000
AP history, n (%)	67 (50.8)	38 (50.00)	0.011	0.916
Months of AP	5.94 ± 3.32	6.39 ± 3.02	–0.696*	0.488
HbA1c, (%)	6.01 ± 1.81	6.49 ± 0.21	–1.759*	0.080
TC, (mmol/L)	5.61 ± 0.62	5.69 ± 0.64	–0.839*	0.403
LDL, (mmol/L)	3.31 ± 0.53	3.35 ± 0.54	–0.553*	0.583
TG, (mmol/L)	1.73 ± 0.10	1.74 ± 0.19	–0.710*	0.487
HDL, (mmol/L)	1.15 ± 0.24	1.19 ± 0.27	–1.064*	0.289
Uric, (µmol/L)	287.76 ± 78.94	288.53 ± 85.67	–0.066*	0.948
Cre, (µmol/L)	61.64 ± 8.91	62.25 ± 13.65	–0.391*	0.904
Hcy, (µmol/L)	9.67 ± 4.02	9.59 ± 4.59	0.120*	0.904

Table 1 note: * is the *t* value. AP, angina pectoris; Cre, creatinine; HbA1c, glycosylated hemoglobin; Hcy, homocysteine; HDL, high-density lipoprotein; LDL, low-density lipoprotein; Months of AP, duration (months) suffered from angina pectoris; Months of diabetes, duration (months) suffered from diabetes; TC, total cholesterol; TG, triglyceride; Uric, uric acid.

### 3.2 Comparison of IRA Distribution

Patients were treated for IRA of the right coronary artery (RCA) 35.58%, left anterior descending branch (LAD) 54.81%, or left circumflex branch (LCX) 9.61%. There were no cases of left main coronary severe stenosis or occlusion. The distribution of IRA was not significantly different between the two groups (*p* < 0.05). See Table 2 for details.

**Table 2.** **Infarct-related artery distribution in study participants**.

	Deferred stenting (n = 132)	Immediate stenting (n = 76)	χ^2^ value	*p* value
RCA, n (%)	41 (31.06)	33 (43.42)	3.215	0.073
LAD, n (%)	79 (59.85)	35 (46.05)	3.706	0.054
LCX, n (%)	12 (9.09)	8 (10.53)	0.114	0.753

Table 2 notes: LAD, left anterior descending branch; LCX, left circumflex branch; RCA, right coronary artery.

### 3.3 Analysis of PCI between Deferred and Immediate Stenting

The IRA thrombus burden score of the 208 STEMI patients ranged from 4–5 points, with an average of 4.50 ± 0.50 points. After PTCA and/or thrombus aspiration, the recalculated thrombus burden score (rescore) in IRA was still ≥4 points (4.43 ± 0.50 in the deferred stenting group and 4.33 ± 0.50 in the immediate stenting group), with no significant difference between the two groups (*p* > 0.05). The diameter of stent implantation in the deferred stenting group was significantly larger than that in the immediate stenting group (*p* ≤ 0.001). The length of stent implantation in the deferred stenting group was significantly shorter compared to the immediate stenting group (*p* < 0.05). See Table 3 for details.

**Table 3.** **Comparative analysis of PCI procedures between deferred and immediate stenting**.

	Deferred stenting (n = 132)	Immediate stenting (n = 76)	*t* value	*p*-value
Thrombus rescore	4.43 ± 0.50	4.33 ± 0.47	1.462	0.145
Thrombus aspiration, n (%)	20 (15.15)	8 (10.53)	0.886*	0.347
Onset to B, (H)	5.45 ± 0.90	5.28 ± 1.21	1.158	0.248
Number of stents	1.49 ± 0.58	1.45 ± 0.53	0.554	0.580
Diameter of stent, (mm)	3.18 ± 0.44	2.93 ± 0.42	3.988	≤0.001
Length of stent, (mm)	15.61 ± 2.80	20.92 ± 6.13	8.425	≤0.001

Table 3 notes: * is χ^2^ value. Onset to B: the time from onset of symptoms to balloon expansion. H, hours; PCI, percutaneous coronary intervention; Thrombus rescore, recalculated thrombus burden score.

### 3.4 Improvements from Deferred Stenting

Following stenting, participants in the deferred stenting group exhibited a significantly lower incidence of distal embolism (3.03%) compared to the immediate stenting group (36.84%, *p* < 0.01). Additionally, deferred stenting was associated with significant increases to both the flow rate of grade 3 TIMI in the IRA and grade 3 myocardial blush when compared to the immediate stenting group (*p* < 0.01). See Table 4 for details.

**Table 4.** **Stenting procedure outcomes**.

	Deferred stenting (n = 132)	Immediate stenting (n = 76)	χ^2^ value	*p* value
Distal embolism, n (%)	4 (3.03)	28 (36.84)	42.537	≤0.001
TIMI flow grade 3, n (%)	130 (98.48)	64 (84.21)	15.654	≤0.001
MBG 3 level, n (%)	65 (98.48)	58 (76.32)	27.275	≤0.001

Table 4 notes: MBG, myocardial blush grade; TIMI, thrombolysis in myocardial infarction.

### 3.5 Clinical Follow-up Outcomes

The follow-up period ranged from 10–14 months, averaging 12.03 ± 1.18 months. Out of the 208 cases, 192 were followed up, yielding a follow-up rate of 92.31% (192/208). There were 16 cases that were lost, primarily due to lost telephone information. The immediate stenting group had a slightly higher follow-up rate (97.37%) compared to deferred stenting group (89.39%), though the reasons for this difference remain unclear (*p* < 0.05). However, the follow-up duration was consistent across both groups (see Table 5).

**Table 5.** **Follow-up outcomes for deferred and immediate stenting**.

	Deferred stenting (n = 118)	Immediate stenting (n = 74)	χ^2^ value	*p* value
Rate of follow-up, n (%)	118 (89.39)	74 (97.37)	4.320	0.038
Follow-up, (months)	11.96 ± 1.26	12.14 ± 1.02	1.017*	0.311
All-cause death, n (%)	3 (2.54)	6 (8.11)	3.153	0.076
Readmission HF, n (%)	2 (1.69)	5 (6.76)	3.317	0.069
Recurrence of MI, n (%)	2 (1.69)	6 (8.11)	4.684	0.030
TLR and/or TVR, n (%)	7 (5.93)	7 (9.46)	0.873	0.360
Repeat PCI, n (%)	8 (6.78)	9 (12.16)	1.633	0.201
MACE, n (%)	11 (9.32)	15 (20.27)	4.656	0.031
LVEF	0.60 ± 0.05	0.58 ± 0.05	3.633*	≤0.001

Table 5 notes: * is the *t* value. LVEF, left ventricular ejection fraction; MACE, major adverse cardiac events; Readmission HF, readmission for heart failure during follow-up; Recurrence of MI, recurrence of nonfatal myocardial infarction; Repeat PCI, repeat percutaneous coronary intervention including TLR and TVR, and any coronary vessel revascularization due to the recurrence of the acute coronary syndrome; TLR, target lesion revascularization; TVR, target vessel revascularization.

Outcomes from the follow-up included the rate of all-cause death, the readmission for heart failure, TLR, and repeat PCI. The deferred stenting group showed a non-significant trend towards decreased values when compared to the immediate stenting group (*p* > 0.05). It’s noteworthy that repeat PCI encompassed both TLR and PCI for any coronary artery lesions and heart failure arising from acute coronary syndrome. No stroke cases were reported during the follow-up period.

Importantly, the LVEF was significantly higher in the deferred stenting group than that in the immediate stenting group (*p* < 0.01). Additionally, the recurrence rate of nonfatal myocardial infarction and MACE in the deferred stenting group were significantly lower in the deferred stenting group compared to the immediate stenting group at the 1-year follow-up (*p* < 0.05).

## 4. Discussion

### 4.1 Deferred Stenting in Geriatric Patients with STEMI and High Thrombosis

The implementation of primary PCI in the treatment of acute coronary occlusion has significantly improved the outcomes for patients with STEMI [17, 18]. Primary PCI is recognized as the standard treatment for patients with STEMI and is most effective when administered within 12 hours from symptom onset to balloon dilatation. Deferred stenting, a novel strategy, involves delaying stent implantation until a stable distal flow is established. However, the efficacy of deferred stenting in STEMI patients with high thrombosis, particularly in geriatric patients, remains a subject of debate.

Our previous research results showed that STEMI patients with high thrombosis in IRA could benefit from deferred stenting [19, 20]. This strategy not only improves myocardial perfusion but also protects cardiac ejection function [19, 20]. Deferred stenting is particularly beneficial for STEMI patients with a substantial thrombus burden. This treatment change may prevent distal embolization, relieve vasospasm, reduce the slow flow or no-reflow phenomena, improve microvascular flow, improve myocardial preservation, attenuate perioperative myocardial infarction, and improve LVEF [19, 20]. Additionally, the deferred strategy allows for more precise stent more precise stent selection [21]. Importantly, the risk of stent mal-apposition and in-stent thrombosis may be reduced with deferred stenting [22, 23, 24, 25]. This reduction is likely due to the avoidance of using smaller-sized stents and longer devices, which can be better assessed and chosen when the urgent phase has passed [22, 23, 24, 25].

This non-randomized controlled trial focused on geriatric patients with thrombus rescore ≥4. The result of the study firstly showed that deferred strategy was beneficial to patients over the age of 80 with STEMI and a heavy thrombus burden. However, the findings from the randomized controlled trials (RCT) have shown inconsistent results compared to the above-mentioned studies. Two highly concerned RCTs are the Minimalist Immediate Mechanical Intervention (MIMI) study and deferred versus conventional stent implantation in patients with ST-segment elevation myocardial infarction (DANAMI 3-DEFER) study [6, 26]. The results of the MIMI RCT study showed that the patients with STEMI did not benefit from delayed stenting [26]. Similarly, the DANAMI 3-DEFER RCT study, an open-label, randomized trial involving 1215 patients equally divided between standard PCI and deferred stent implantation, found that routine deferred stenting did not significantly improve outcomes [6]. Over a median follow-up of 42 months, the study observed no notable difference in the incidence of death, heart failure, myocardial infarction, or repeat revascularization between the standard PCI and deferred stenting groups [6]. Additionally, Procedure-related myocardial infarction, bleeding requiring transfusion or surgery, contrast-induced nephropathy, or stroke were similar across both groups, occurring in 28 (5%) patients in the conventional PCI group and in 27 (4%) patients in the deferred stent implantation group, with no significant differences between groups [6].

The results of a comparative meta-analysis [9] including 1456 patients with STEMI across three randomized controlled trials and 719 patients with STEMI in six observational studies showed that compared with immediate stenting, a deferred-stenting strategy did not reduce the occurrence of no- or slow-reflow, death, myocardial infarction, or repeat revascularization. However, the results did show a long term improved left ventricular function.

In contrast to previous results, Nepper-Christensen, *et al*. [27] reported angiographic outcomes in patients treated with deferred stenting after STEMI. A total of 1205 patients with STEMI were randomized to deferred (n = 594) versus immediate stent implantation (n = 611) [27]. The results showed a lower incidences of distal embolization (odds ratio [OR] = 0.67, 95% confidence interval [CI]: 0.46–0.98, *p* = 0.040) and slow/no-reflow (OR = 0.60, 95% CI: 0.37–0.97, *p* = 0.039). In high-risk subgroups, the protective effect was greatest in patients >65 years of age (slow/no-reflow: OR = 0.36, 95% CI: 0.17–0.72, *p* = 0.004, and distal embolization: OR = 0.34, 95% CI: 0.18–0.63, *p* = 0.001). The results indicate that deferred stent implantation reduced the incidences of slow/no-reflow and distal embolization, especially in older patients and in those with total coronary occlusion or a high level of thrombus burden.

The divergent outcomes observed in studies investigating deferred stenting for STEMI patients are primarily attributed to differences in study design, particularly in the selection of subjects and the timing of the deferred intervention. In randomized trials, the critical factor of thrombus burden is often overlooked. Patients, regardless of their high thrombus burden, are randomly assigned to either the delayed or immediate stenting groups. Moreover, the deferred period in these trials, ranging from 24 to 72 hours, was too short to allow sufficient time for thrombus dissolution to disappear.

The results of the aforementioned RCTs indicate that deferred stenting does not offer benefits for non-selected STEMI patients, but may be advantageous for the specific subgroup of STEMI patients with high thrombus burden. In our study, the decision to perform stent implantation during PCI was at the discretion of the interventional operator. Consequently, in some cases within the immediate stenting group, the operator had to promptly proceed with stent implantation due to early recoil of the IRA and/or flow-limiting dissection. This situation could have introduced a minor deviation relating the thrombus burden and blood flow level in IRA. However, this phenomenon did not affect the results of the study, as the subjects of the study were the selected STEMI patients with a high thrombotic burden, and only a few suffered from a flow-limiting dissection. The rescore of thrombus burden (4.43 ± 0.50) in the deferred stenting group was not significantly different from the immediate stenting group (4.33 ± 0.47, *p* > 0.05). The result suggests that the assessment of thrombus burden in patients in the immediate stenting group may not have been affected. The results of this study showed that elderly STEMI patients with high thrombus burden can benefit from the deferred strategy, which was consistent with the outcomes of most observational studies [19, 20, 21, 24, 25, 26, 27, 28, 29].

Recent studies have confirmed the idea that deferred stenting is beneficial to STEMI patients with high thrombus burden [28, 29]. For these patients, re-evaluating thrombus burden after achieving TIMI flow grades 2–3 through PTCA using balloons (1.5 × 15 mm or 2.0 × 20 mm) and/or thrombus aspiration is crucial. If the thrombus burden rescores are still ≥4, deferred stenting can be beneficial to STEMI patients, performed after continuing antithrombotic (dual antiplatelet and anticoagulation) treatment for 7–8 days. However, it is not clear at present that geriatric patients (age ≥80) with STEMI and high thrombus burden also benefit from deferred stenting.

Our study recruited 208 STEMI patients (age 80–87 years) with high thrombus burden in the IRA. After PTCA (with 1.5 × 15 mm or 2.0 × 20 mm balloon) and/or thrombus aspiration, the rescore of residual thrombus burden in IRA was still ≥4 points (4.43 ± 0.50 in the deferred stenting group vs 4.33 ± 0.47 in the immediate stenting group, *p* > 0.05). The results of the study showed that deferred stenting helped to reduce the distal embolization rate, improve the rate of TIMI blood flow grade 3 and myocardial blush grade 3, protect the ejection function of the heart, decrease the rate of MACE, and decrease the recurrence of nonfatal myocardial infarction (See Table 5). It has been confirmed that treatment with deferred stenting was safe and effective in geriatric patients with both STEMI and high thrombus burden. This outcome may be critically important to clinical practice.

In the study, the rate of all-cause death, readmission for heart failure, TLR, and repeat PCI in the deferred strategy was numerically lower than that in the immediate stenting strategy but did not reach statistical significance (*p* > 0.05) at the 1-year follow-up. Our results are consistent with the study from Cassese *et al*. [30]. MACE is affected by many variables, including the management of lipids, blood pressure, blood sugar, tobacco control, lifestyle, compliance of patients with antithrombotic and anti-arteriosclerosis treatment, and so on. A meta-analysis (including one randomized and five observational studies) demonstrated that deferred stenting was safe and effective, and had a lower MACE rate [31]. The result of the study suggests that for STEMI patients with a high thrombus burden, delayed stenting should be performed after 7–8 days of continuous antithrombotic therapy, if the thrombus burden rescore remains high after PTCA and/or thrombus aspiration. The results of the SUPER-MIMI study [32] showed that deferred stenting (deferral time ≥7 days) was beneficial and safe for STEMI patients with a high thrombus burden. Furthermore, the TIMI flow was maintained or improved upon between the end of the first procedure and the beginning of the second procedure in all patients. Overall, thrombotic burden and stenosis severity diminished significantly between the two procedures.

### 4.2 What is the Ideal Deferral Time?

Most researchers agree that the ideal delay for deferred stenting, yielding favorable outcomes, is around 7–8 days [7, 24, 32]. However, in four RCTs—DEFER STEMI [33] (delay 4–16 h), MIMI [26] (delay 24–48 h), DANAMI 3-DEFER [6] (delay 48 h), and a Danish pilot study [34] (delay 48–72 h)—the deferred time was much shorter. These RCTs concluded that deferred stenting did not show superiority to immediate stenting for patients with STEMI regardless of thrombus burden in the IRA.

In contrast, the INNOVATION RCT [35] (delay 3–7 days) and three observational studies—SUPER-MIMI [32] (delay 7–12 days), Tang *et al*. [24] (delay 7 days) and Ke *et al*. [7] (delay 7 days)—adopted a longer delay. These studies found that clinical outcomes were improved with deferred stenting compared to immediate stenting when the delay was around 7 days. Many researchers, including our group, consider the delay window of 24–48 h or 48–72 h too brief for substantial thrombus resorption and effective action of antithrombotic agents. A delay that’s too prolonged risks extended ischemic injury, while a too-short wait may lead to overly aggressive reperfusion that will lead to reperfusion injury. This necessitates a “Goldilocks” approach to determine the optimal stenting time (not too long and not too short) to achieve an optimal outcome [36].

In this study, we observed that stents used in the deferred stenting group were significantly larger in diameter but shorter in length compared to those in the immediate stenting group (*p* < 0.05). This outcome suggests that during the 7–8 day period of antithrombotic therapy, the thrombus naturally dissolves, the vasospasm is relieved, TIMI blood flow improves, and the risk of slow flow or no-reflow are reduced. Consequently, the true dimensions of the vascular lumen diameter and length of the IRA lesion can be measured with greater accuracy. This precision enables the avoidance of selecting stents that are too small in diameter or excessively long.

Supporting this, Harbaoui, *et al*. [21] also found that deferred stenting resulted in the use of stents with larger diameters and shorter lengths compared to immediate stenting. This likely reflects the more accurate lesion assessment possible after the relief of spasm and thrombus resolution during the delay. Such precise stent selection may reduce the likelihood of in-stent restenosis. This interpretation is supported by the other results demonstrating that the incidence of in-stent restenosis is positively correlated with the length of the implanted stent and negatively correlated with the diameter of the implanted stent [37, 38].

### 4.3 How Should Antithrombotic Therapy be Performed during the Delay Period of Deferred Stenting?

Antithrombotic therapy is the cornerstone of treating STEMI patients, with the primary goal of achieving effective anti-ischemic effects while minimizing bleeding risks. In patients with STEMI and high a thrombus burden, even after restoring stable blood flow in the IRA through PTCA or thrombus aspiration, there is still a heavy thrombus in IRA. This necessitates ongoing antithrombotic treatment until the second PCI. The purpose of antithrombotic therapy is to prevent new thrombosis, consolidate stable blood flow, and promote the automatic dissolution and disappearance of thrombi. A continuous antithrombotic regimen, typically lasting 7–8 days, allows the gradual dissolution and eventual disappearance of the thrombus under the influence of blood flow. A shorter duration of antithrombotic therapy may not suffice for the spontaneous resolution of the thrombus in the IRA. The antithrombotic therapy should include using anticoagulant drugs such as heparin sodium or bivalirudin, in addition to the standard dual antiplatelet therapy (DAPT).

In this study, deferred stenting was performed after 7–8 days (mean 7.11 ± 0.32 days) of continuous antithrombotic therapy, which included standard DAPT and anticoagulation. Specifically, patients in the deferred stenting group received subcutaneous injections of heparin sodium at 100 U/kg every 8 hours for the 7–8-day period leading up to the next PCI. After the PCI procedure, all patients were administered antithrombotic drugs, including low molecular weight heparin (enoxaparin sodium 1.0 mg/kg subcutaneously every 12 hours for 3 days) and continued on DAPT (maintenance doses of aspirin 100 mg/d and clopidogrel 75 mg/d, oral administration for at least one year).

A study by Magdy, *et al*. [39] reported that 150 patients with STEMI were randomly divided into three groups: early deferral group (Group A, n = 50, 4–16 h later), late deferral (Group B, n = 50, after 7 days), and immediate stenting (Group C, n = 50). For deferred stenting, the antithrombotic strategy included a continuous intravenous infusion of glycoprotein IIb/IIIa inhibitor for 48 h (irrespective of time of deferral of stenting), subcutaneous low molecular weight heparin (enoxaparin 1 mg/kg every 12 h until the 2nd procedure), and DAPT with aspirin and ticagrelor [39]. Their findings showed a significant improvement in thrombus resolution in group B (deferral 7 days) compared to group A (deferral 4–16 hours, *p* < 0.001) along with improvements in other clinical outcomes compared to groups A and C [39]. Furthermore, the HORIZONS-AMI study [40] found that in STEMI patients undergoing PCI, bivalirudin reduced the risk of major bleeding and cardiac death compared to heparin. The increased risk of bleeding in the heparin group is believed to be associated with the simultaneous use of platelet glycoprotein IIb/IIIa inhibitors (GPI). When used alongside GPI, the heparin dosage should be halved to mitigate bleeding risks. In this study, all patients received standard DAPT with maintenance doses of aspirin (100 mg/d) plus clopidogrel (75 mg/d) for at least one year after stenting procedure, with no acute thrombotic or bleeding events reported.

The challenge in DAPT lies in balancing the reduction of ischemic risk against the increased risk of bleeding. Addressing this ‘Goldilocks dilemma’, where too short a DAPT duration raises ischemic events and too long increases bleeding risk, the TWILIGHT-COMPLEX study [41] proposed an approach for complex PCI patients. Initially, DAPT was administered for three months, followed by ticagrelor monotherapy for 12 months or more. The study found that ticagrelor alone did not significantly increase MACE involving ischemic or hemorrhagic incidents compared to traditional double antiplatelet therapy (Aspirin + P2Y12 inhibitor [platelet adenosine diphosphate receptor subunit 12 inhibitor]) (*p* > 0.05). Furthermore, the aggregated results of four RCTs involving 29,089 PCI patients [42] revealed that after implanting a current drug-eluting stent, transitioning from standard DAPT to P2Y12 inhibitor monotherapy resulted in a lower incidence of clinically relevant bleeding compared to maintaining DAPT for 12 months. This change did not lead to significant differences in major adverse cardiac or cerebrovascular events at the one-year mark. These findings suggest that patients undergoing complex PCI might benefit from switching to P2Y12 inhibitor monotherapy after an initial three months of DAPT.

## 5. Limitations of This Study

Geriatric patients (aged ≥80 years old) with STEMI and high thrombus burden in IRA represent a distinct and relatively small population, resulting in a limited sample size for this study. To validate our findings, further research with larger sample sizes is necessary. While our results have indicated that deferred stenting may benefit octogenarians with STEMI and a high thrombus burden in IRA, there remain several minor problems that need addressing in future studies.

In this study, all subjects were STEMI patients who underwent emergency PCI, precluding the possibility of a cardiac ultrasound prior to PCI to assess cardiac function. Therefore, LVEF was only compared between the two groups at the last follow-up. The indicator of thrombus evaluation used in this study was based on CAG imaging and was not based on intravascular ultrasound or optical coherence tomography, which might lead to inaccuracies in the estimation of the thrombus volume in the IRA. Due to the lack of continuous monitoring of serum creatine kinase (CK) values, the difference in CK peak values of the two groups were not compared. In the deferred stenting group, anticoagulant therapy was not monitored by ACT from the first PCI to second PCI, which could affect the identification of anticoagulant efficacy. Additionally, the differing follow-up rates between the groups suggest that these results require validation by larger, more comprehensive studies.

## 6. Conclusions

In the deferred stenting group, compared to the immediate stenting group, there were notable benefits: larger diameters and shorter lengths of stent implantation, a lower rate of distal embolism in the IRA, higher rates of TIMI blood flow grade 3 and myocardial blush, better LVEFs at the 1-year follow-up, and a lower MACE rate. These outcomes suggest that deferred stenting enhances the precision of stent implantation. For geriatric patients (aged ≥80 years) with STEMI and a high thrombus burden, deferred stenting not only diminishes the rate of distal embolism in the IRA but also improves myocardial tissue perfusion, protects cardiac ejection function, and demonstrates good safety.

## Data Availability

All data generated or analyzed during this study are included in this published article. Data other than these are available from the corresponding author upon reasonable request.

## Abbreviations

ACT, activated clotting time of whole blood; AP, angina pectoris; CABG, coronary artery bypass grafting; CAG, coronary angiography; CK, creatine kinase; Cre, creatinine; DAPT, dual antiplatelet therapy; HB, hemoglobin; HbA1c, glycosylated hemoglobin A1c; Hcy, Homocysteine; HDL, high-density lipoprotein; IRA, infarct-related artery; LAD, left anterior descending branch; LCX, left circumflex branch; LDL, low-density lipoprotein; LVEF, left ventricular ejection fractions; MACE, major adverse cardiac events; MBG, myocardial blush grade; PCI, percutaneous coronary intervention; PGI, platelet glycoprotein IIb/IIIa inhibitors; PTCA, percutaneous transluminal coronary angioplasty; RCA, right coronary artery; RCT, randomized controlled trial; STEMI, ST-segment elevation myocardial infarction; TC, total cholesterol; TG, triglyceride; TIMI, thrombolysis in myocardial infarction; TLR, target lesion revascularization; TVR, target vessel revascularization; Uric, uric acid.
